# Extracorporeal Shock Wave Therapy Combined with Complex Decongestive Therapy in Patients with Breast Cancer-Related Lymphedema: A Systemic Review and Meta-Analysis

**DOI:** 10.3390/jcm10245970

**Published:** 2021-12-19

**Authors:** Yu Lin Tsai, Ting Jie I, Ya Chi Chuang, Yuan Yang Cheng, Yu Chun Lee

**Affiliations:** 1 Department of Physical Medicine and Rehabilitation, Taichung Veterans General Hospital, Taichung 40705, Taiwan; u100001304@cmu.edu.tw (Y.L.T.); s851075@ym.edu.tw (Y.Y.C.); 2 Department of Medical Education, Taichung Veterans General Hospital, Taichung 40705, Taiwan; inty114@vghtc.gov.tw (T.J.I.); u103001313@cmu.edu.tw (Y.C.C.); 3 School of Medicine, National Yang Ming Chiao Tung University, Taipei 11221, Taiwan

**Keywords:** ESWT, extracorporeal shock wave therapy, BCRL, breast cancer, lymphedema, systemic review and meta-analysis

## Abstract

Breast cancer-related lymphedema (BCRL) is one of the most significant complications seen after surgery. Several studies demonstrated that extracorporeal shock wave therapy (ESWT), in addition to conventional complex decongestive therapy (CDT), had a positive effect on BCRL in various aspects. The systematic review and meta-analysis aim to explore the effectiveness of ESWT with or without CDT on BRCL patients. We searched PubMed, Embase, PEDro, Cochrane Library Databases, and Google Scholar for eligible articles and used PRISMA2020 for paper selection. Included studies were assessed by the PEDro score, Modified Jadad scale, STROBE assessment, and GRADE framework for the risk of bias evaluation. The primary outcomes were the volume of lymphedema and arm circumference. Secondary outcome measures were skin thickness, shoulder joint range of motion (ROM), and an impact on quality-of-life questionnaire. Studies were meta-analyzed with the mean difference (MD). Eight studies were included in the systemic review and four in the meta-analysis. In summary, we found that adjunctive ESWT may significantly improve the volume of lymphedema (MD = −76.44; 95% CI: −93.21, −59.68; *p* < 0.00001), skin thickness (MD = −1.65; 95% CI: −3.27, −0.02; *p* = 0.05), and shoulder ROM (MD = 7.03; 95% CI: 4.42, 9.64; *p* < 0.00001). The evidence level was very low upon GRADE appraisal. ESWT combined with CDT could significantly improve the volume of lymphedema, skin thickness, and shoulder ROM in patients with BCRL. There is not enough evidence to support the use of ESWT as a replacement for CDT. This study was registered with PROSPERO: CRD42021277110.

## 1. Introduction

The worldwide prevalence rate of breast cancer has increased continuously since 1990, whereas the overall mortality rate has decreased in most high-income countries [1,2]. In other words, an increasing number of breast cancer survivors are suffering from complications brought on by surgery. Breast cancer-related lymphedema (BCRL) is one of the most significant complications seen after surgery. It could happen at any time post-operation with an overall incidence rate of 21.4% [3] but varies from 5% to 60% due to various diagnostic criteria [3,4]. Factors that can increase the risk of BCRL include the number of lymph nodes removed, axillary radiotherapy, a high body mass index, and cellulitis [5,6]. Since BCRL remains both incurable and debilitating [7], the excessive lymph fluid accumulation in the upper limb may cause lifelong functional, aesthetic, and psychological problems [8]. Conventionally, non-invasive complex decongestive treatments (CDT) for BCRL include manual lymph drainage, intermittent pneumatic compression, compression bandage or garments, regular exercise, and skin care [9]. Although a decrease of 50–70% in lymphedema volume through CDT has been reported [10], its effectiveness depends largely upon each therapist’s experience and overall patient compliance [10,11,12]. Furthermore, in patients diagnosed with stage two or three lymphedema, local fibrotic change may also alternate the effect of CDT [9,13] and force patients to seek additional treatment.

Extracorporeal shock wave therapy (ESWT) has been used as a physical therapy modality over the past decades [14,15]. Shockwave therapy can be delivered to tissues by two distinct mechanisms: focused shockwave therapy and radial shockwave therapy. The two types of shockwaves differ in their depth of tissue penetration, ability to focus the shockwave, and the rapidity of the rise and fall of pressure (shape of the shockwave). Generally, a focused shockwave is a single pulse with a wide frequency range (from approximately 150 kHz up to 100 MHz), high pressure amplitude (up to 150 MPa), low tensile wave (up to −25 MPa), small pulse width, and a short rise time of up to a few hundred nanoseconds [16,17]. On the other hand, in a radial shockwave, the energy is dissipated over a large area, which reaches maximal pressure at the source instead of selected depths in tissues. The intensity at the focal point of the focused shockwave, which is measured as energy flux density (EFD; mJ/mm^2^) per impulse, ranging from 0.001 to 0.5 mJ/mm^2^, could be further classified as low energy and high energy with 0.2 mJ/mm^2^ as the cutoff point [18]. As for the radial shockwave, the pressure unit (bar) is often mentioned instead of energy flux density, and the conversion from radial to focused type can be completed [19]. Plenty of available research has proved that low-energy ESWT helps tissue regeneration by increasing stem cell activity, promoting endothelial neoangiogenesis, regulating inflammation, relieving pain, and preventing soft tissue fibrosis [20,21,22]. Regarding lymphatic tissue, in vivo and in vitro studies have demonstrated that ESWT could change both gene regulation and mRNA expression in endothelial cells, ultimately leading to lymphangiogenesis [23,24]. Michelini et al. [25] first used ESWT as a form of treatment for primary and secondary lymphedema of the upper and lower limbs, and observed a 32% reduction in the circumference of the affected limbs, as well as less consistency in fibrotic areas.

More recently, several randomized controlled trials (RCTs) and clinical studies have been conducted to compare the effectiveness of low-energy ESWT together with CDT in patients with BCRL. Bae et al. [26] found that ESWT reduced the volume, circumference, and skin thickness of patients’ arms with lymphedema. Cebbici et al. [27] also demonstrated that ESWT improved both functional status and quality of life in breast cancer patients. Although most of these investigations affirmed ESWT as being an effective management tool for BCRL, because outcome measures and methods varied, it was difficult to assert the significance of ESWT’s therapeutic effect for BCRL.

Up until now, we had found only two narrative literature reviews [28,29] discussing the use of ESWT on BCRL, while comprehensive systemic review and meta-analysis are still lacking. As a result, the objectives of this article are to identify that:

1. Does adjunctive ESWT provide additional benefits for BCRL patients compared to CDT alone? If so, in what manner?
2. Can ESWT serve as a replacement for CDT in patients with BCRL? If so, in what aspect?

## 2. Materials and Methods

Our study was registered in the International System Review Prospective Register (PROSPERO) (CRD42021277110) and conducted according to the Preferred Reporting Items for Systematic Reviews and Meta-Analyses (PRISMA) statement published in 2020 [30]. Because this study did not involve any clinical intervention or processing of individual patient data, Institutional Review Board (IRB) approval was unnecessary.

### 2.1. Identification and Selection of Studies

We searched PubMed, Embase databases, Physiotherapy Evidence Database (PEDro), Cochrane Library Databases, and Google Scholar for any relevant publications up until September 2021. Different combinations of the following keywords and MeSH terms were used in our initial literature search: “breast cancer”, “lymphedema”, “edema”, “ESWT”, and “shockwave”. Standard Boolean operators (AND, OR) were applied to link the terms, as shown in Table S1. The reference lists of selected articles were also hand-searched for additional related studies.

Only titles and abstracts from the search results were reviewed by two co-authors (Y.L.T. and Y.C.C.), with the results, authors, and journal titles being blinded. Studies were considered eligible if they met the inclusion criteria in Table 1, with full texts being reviewed thoroughly, including the reference list for additional related articles. The two reviewers worked independently, and any discrepancies were resolved through discussion and consensus involving a third reviewer (Y.C.L.).

### 2.2. Assessment of the Characteristics of Studies

#### 2.2.1. Quality

Reviewers (Y.L.T. and Y.C.C.) independently performed quality validity and critical appraisal of the included trials through the use of the Cochrane Risk of Bias Tool [31], PEDro scale score [32], and Modified Jadad Scale [33]. According to the Cochrane Risk of Bias Tool, trials were classified as being high risk if they met less than five of the eight criteria. The PEDro scale score consisted of 11 items, each valued at 1 point if qualified. The higher the total score, the better the methodological quality. Similarly, the Modified Jadad Scale had eight items/points, and studies with 4 to 8 points being considered good to excellent quality.

For non-randomized clinical trials, the Strengthening the Reporting of Observational studies in Epidemiology (STROBE) assessment tool [34] was applied. The number of qualified items was in proportion to the methodological quality of the corresponding study.

#### 2.2.2. Participants

Participants in the included studies involved patients with BCRL.

#### 2.2.3. Intervention

The protocol of ESWT was recorded as intervention: type of ESWT (focused or radial); treatment location, frequency, and intensity; the total number of treatment sessions. We also documented the use of CDT, with or without ESWT, in both the experimental and control groups.

#### 2.2.4. Outcome Measures

The primary outcomes of our review were the decrease in volume of lymphedema and circumference of the affected limb. The secondary outcomes were the skin thickness at fibrotic areas, increase of shoulder joint range of motion (ROM), and the results from the quick Disability of the Arm, Shoulder, and Hand (qDASH) questionnaire [35]. Studies needed to consist of at least one of these results to be included.

#### 2.2.5. Data Extraction and Data Analysis

Name of the author, year of publication, the country where the study was conducted, number of patients, characteristics of patients, therapeutic protocol, and outcomes mentioned above were all extracted if possible. We contacted the authors by email for any missing data and other uncertain issues, which would subsequently be marked as N/A or unclear if we received no response. A study would not be included in the meta-analysis if the necessary data were inaccessible. Based on the Cochrane Handbook [31], continuous data were expressed as the mean ± standard deviation and summarized as a Mean Difference (MD) or Standardized Mean Difference (SMD). We used Cochran’s chi-squared test (Q test) and the I-squared test to assess statistical heterogeneity. All results were reported with 95% confidence intervals (CIs), with *p* < 0.05 considered statistically significant. A fixed-effects model was used when no obvious statistical heterogeneity existed (I^2^ value < 50%); otherwise, a random-effects model was applied (I^2^ value > 50% and *p* < 0.01). Between-group comparison was conducted if enough data from the RCTs (≧2 studies) could be acquired. In case of high statistical heterogeneity (I^2^ value > 75%), sensitivity analysis or funnel plot would be performed if the number of trials sufficed. We performed the systemic review and meta-analysis with Review Manager software (version 5.4; Cochrane Collaboration, London, UK) for graphical representation of the pooled data.

We adopted the Grading of Recommendations Assessment, Development and Evaluation (GRADE) [36,37] framework to assess the intergroup certainty of evidence.

## 3. Results

### 3.1. Study Selection Process

**Figure 1:**
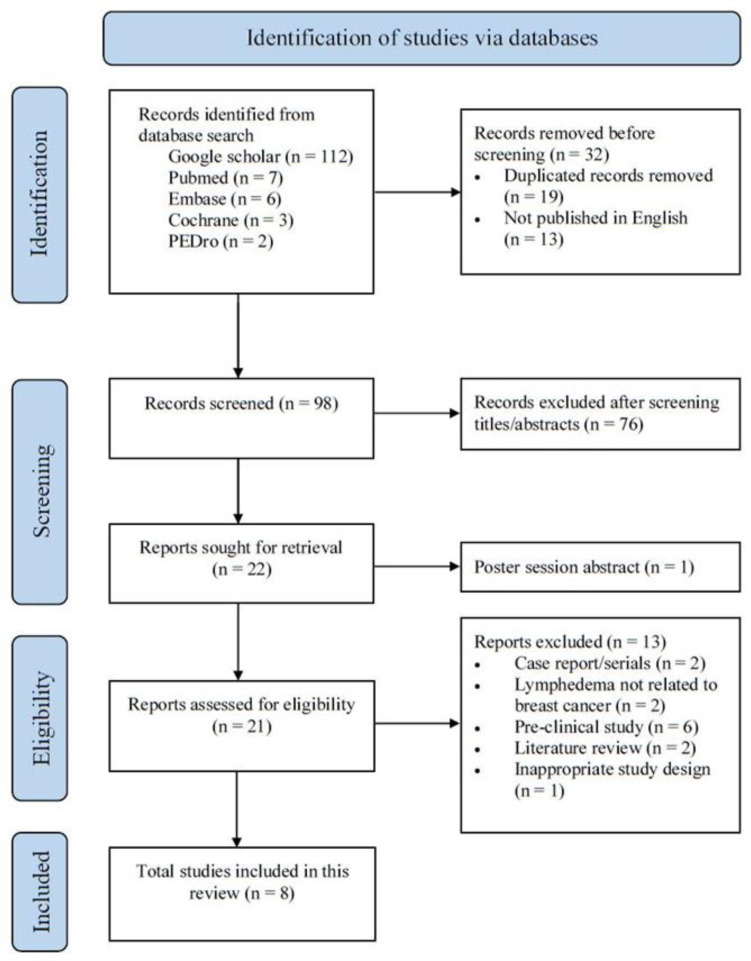
PRISMA2020 flow chart showing literature search and selection process.

One hundred and thirty (130) papers were initially identified using our search strategy. Publication titles were screened, and any duplicates taken from different databases were removed. Twenty-one (21) potentially eligible trials were retrieved for full-text article screening. Of these, only eight studies met all the established criteria and were thus assessed. The selection process is shown in a PRISMA flow chart (Figure 1). Search strategy and reason for exclusion are presented in Tables S1 and S2.

### 3.2. Characteristics of Selected Studies

Six studies were RCTs [38,39,40,41,42,43], of which four originated from Egypt [38,39,40,41], one from South Korea [42], and one from Turkey [43]. Notably, two studies were conducted by the same authors during the same period (El-Shazly et al., 2016 [39]; El-Shazly et al., 2016 [40]) with identical group sizes. We contacted the corresponding authors for clarification but received no reply. Because outcome measures differed, we still considered them as being different studies, but included only one for size pooling.

Another two eligible clinical trials were prospective pilot studies from Turkey [27] and Belgium [44]. One (Cebicci et al., 2016 [27]) was the pilot study of another RCT (Cebicci et al., 2021 [43]) and was therefore exempted from the meta-analysis.

The characteristics of these studies are summarized in Table 2.

#### 3.2.1. Quality

**Figure 2:**
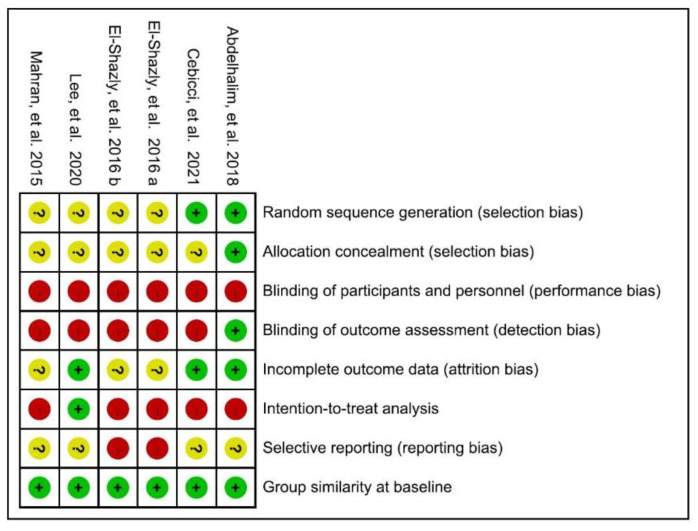
Cochrane risk of bias summary for the included articles. Mahran et al., 2015 [38]; Ely-Shazly et al., 2016 a [39] b [40]; Abdelhalim et al., 2018 [41]; Lee et al., 2020 [42]; Cebicci et al., 2021 [43].

One RCT was considered at low risk of bias [41], while the remaining five trials were rated as high risk, as shown in Figure 2. According to their PEDro scores, two [41,42] of the six included RCTs were considered to be of good methodological quality (33%, scored 6–7), with the rest being of fair quality (67%, scored 4–5). When using the Modified Jadad Scale involving eight items for quality appraisal, half [41,42,43] of the RCTs were considered high quality (50%, scored 5–6.5) while the others were of low quality (50%, scored 3).

We used STROBE assessment tools for the two non-RCT studies. They [27,44] both met the 65.6% (21/33) quality items threshold and were therefore considered to be of fair quality. Quality ratings for each study are presented in Tables S3–S5.

#### 3.2.2. Participants

Of the six RCTs, the largest number of participants was 60 (El-Shazly et al., 2016 [39]), while the lowest was 20 (Cebicci et al., 2021 [43]). The pooled size total was 193 (CDT with ESWT, *n* = 86; CDT alone, *n* = 97; ESWT alone, *n* = 10), with one RCT (El-Shazly et al., 2016 [40]) excluded for reasons previously outlined. In prospective pilot studies, one study recruited 11 patients for ESWT, and the other involving 10 patients for ESWT plus CDT.

#### 3.2.3. Intervention

While five RCTs [38,39,40,41,42] compared the effectiveness of ESWT plus CDT, only one (Cebicci et al., 2021 [43]) investigated the effect of ESWT versus CDT. Half of these studies used radial ESWT on the axillary and cubital lymph nodes and the whole affected upper limb, while the three remaining studies involved focused ESWT on the most fibrotic area and cubital lymph nodes. The treatment was performed two to three times per week for a maximum of 16 sessions. The therapeutic protocols of the included trials are summarized in Table 3.

#### 3.2.4. Outcome Measures

Used in six trials, volume displacement was the most commonly used outcome measure. Five studies [27,38,39,42,43] used the water-displacement method to measure the volume of lymphedema; only one [44] used infrared perometry. Four studies [27,38,42,43] measured the difference in arm volume between the healthy and lymphedema sides; the remaining two studies [39,44] only measured the volume of the affected arm.

Arm circumference was the second most frequently used measure, as employed in five of the studies. Mahran et al. [38] compared the summation of each side arm circumference from multiple levels, while another two studies [41,43] measured the difference between the healthy and affected arms at multiple levels. The remaining two trials [42,44] measured several sites from only the lymphedema side.

Both skin thickness and the qDASH questionnaire were exploited in three of the studies. Two studies [41,42] used a skinfold caliper to measure skin thickness, while the other used sonography [40] as a measurement tool. Joint ROM was performed in two studies.

The outcome measures are summarized in Table 4. GRADE framework was used for intergroup outcome measure comparison, as shown in Table S6. Most studies indicated a positive effect resulting from ESWT, with none reporting any risks or adverse effects following the intervention.

### 3.3. Effect of Intervention

The summarized un-pooled data of primary outcomes can be found in Table 5 and Table 6. The summarized un-pooled data of secondary outcomes can be found in Tables S7 and S8.

#### 3.3.1. Primary Outcome: Volume of Lymphedema

**Figure 3:**
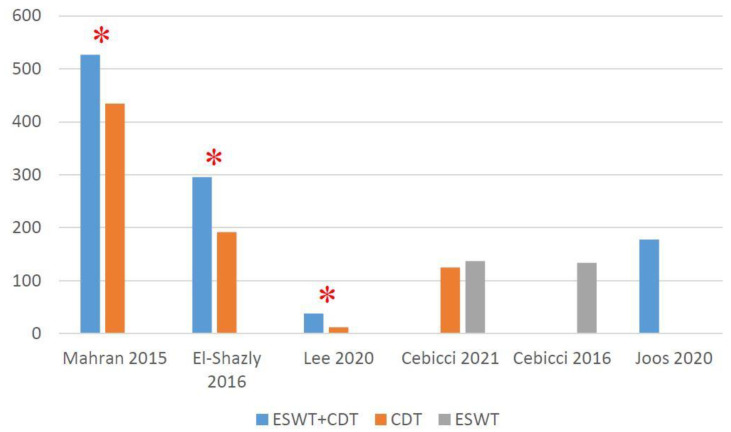
Absolute volume change (mL) from baseline at post-treatment. * Significant inter-group difference.

Three RCTs [38,39,42] narrated the significant effectiveness of the additional ESWT when compared with CDT alone. One RCT [43] showed the significant effect of both ESWT and CDT, although no significant intergroup difference was found. In the two prospective pilot studies, one [27] showed the significant effect of ESWT, while the other [44] revealed no significant effect of additional ESWT with CDT. The mean volume difference between baseline and post-treatment is shown in Figure 3.

**Figure 4:**
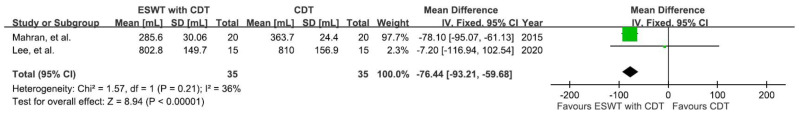
Mean difference (95%CI) in the effect of additional ESWT compared with CDT alone on the volume of BCRL.

Subjects from two RCTs [38,42], which consisted of four groups, were assessed for the effectiveness of ESWT on decreasing the volume of lymphedema (Figure 4). The difference in bilateral upper-limb size in the experimental groups (ESWT plus CDT) and control groups (CDT) was recorded after an intervention. Using a fixed-effect model, the result showed a significant reduction in post-intervention lymphedema volume in the experimental groups compared to the control groups (MD = −76.44; 95% CI: −93.21, −59.68; *p* < 0.00001; I^2^ = 36%). However, the certainty of the evidence was quite low.

#### 3.3.2. Primary Outcome: Arm Circumference

**Figure 5:**
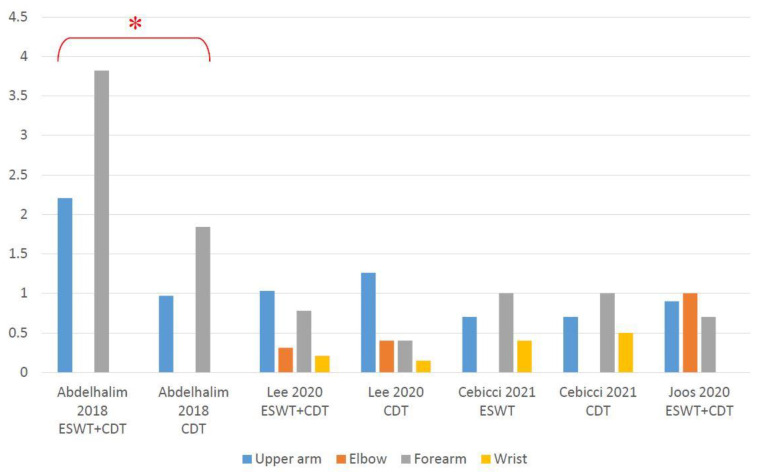
Absolute arm circumference change (cm) from baseline at post-treatment. * Significant inter-group difference.

Two RCTs [38,41] demonstrated significant effectiveness of employing additional ESWT when compared to CDT on its own. Lee et al. [42] revealed a reduction in the circumference at 10 cm below the elbow in their experimental group. Cebicci et al. [43] showed a significant reduction in arm circumference in both groups, except at 10 cm below the elbow and wrist in the control group, with no intergroup difference being found in either. On the contrary, results from Joos et al. [44] showed no obvious circumference decrease after using ESWT plus CDT. The mean arm circumference difference between baseline and post-treatment showed in Figure 5. Detailed data can be seen in Table S9.

#### 3.3.3. Secondary Outcome: Skin Thickness

Three studies [40,41,42] showed a significant skin-thickness reduction in the experimental group (ESWT plus CDT) compared to the control group (CDT only).

**Figure 6:**
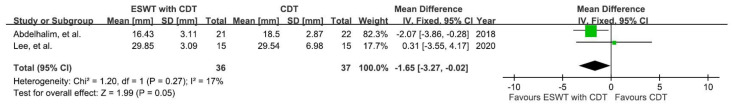
Mean difference (95%CI) in the effect of additional ESWT compared with CDT alone on the skin thickness of BCRL.

The thickness of most fibrotic skin was measured using a skinfold caliper after the intervention. Subjects involved in two studies that included four groups [41,42] were recruited for meta-analysis (Figure 6) using a fixed-effect model. The results showed a significant reduction in post-intervention skin thickness in the experimental groups compared to the control groups (MD = −1.65; 95% CI: −3.27, −0.02; *p* = 0.05; I^2^ = 17%); however, the certainty of pooled data was very low.

#### 3.3.4. Secondary Outcome: Shoulder Range of Motion

**Figure 7:**
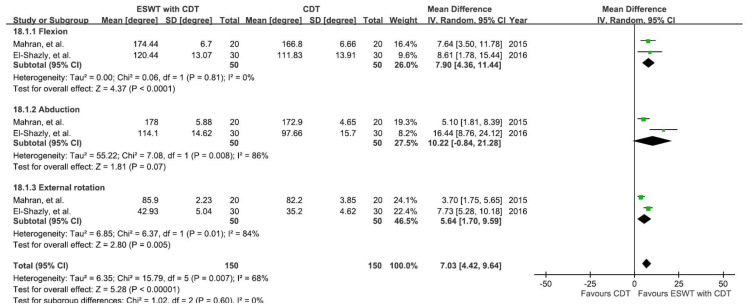
Mean difference (95%CI) in the effect of additional ESWT compared with CDT alone on the shoulder ROM of BCRL.

The results from two studies involving six groups [38,39] were analyzed to see the effects on both the experimental group (ESWT plus CDT) and control group (CDT alone) in improving shoulder joint ROM, as shown in Figure 7. The ROM of shoulder flexion, abduction, and external rotation were measured before and after the intervention.

A random-effect model was applied, showing a significant improvement (MD = 7.03; 95% CI: 4.42, 9.64; *p* < 0.00001; I^2^ = 68%) in shoulder joint ROM in the experimental groups compared to the control groups. Subgroup analysis further showed a significant ROM improvement in flexion (MD = 7.90; 95% CI: 4.36, 11.44; *p* < 0.0001; I^2^ = 0%) and external rotation (MD = 5.64; 95% CI: 1.70, 9.59; *p* = 0.005; I^2^ = 84%) in the experimental groups versus the control group. However, the certainty was very low, and sensitivity analysis could not be performed due to the lack of a sufficient number of trials.

#### 3.3.5. Secondary Outcome: qDASH

In one study (Cebicci et al. [27]), patients reported a better qDASH score after receiving ESWT for BCRL. The following RCT also demonstrated that patients from both the experimental and the control groups had improved qDASH score after the intervention, with the former to a greater degree [43]. However, Lee et al. [42] found no difference between pre- and post-intervention qDASH scores in both groups. Detailed data were summarized in Table S10.

The meta-analysis of qDASH was not conducted due to insufficient data.

## 4. Discussion

According to our knowledge and data taken from literature searches, this article is the first formal systemic review and meta-analysis comparing the use of additional ESWT, versus only conventional CDT, on multiple outcomes for BCRL patients based upon RCTs and observational studies. In this study, we found a significant improvement in arm volume, skin thickness, and shoulder joint ROM between the experimental and control groups (Figure 4, Figure 6, and Figure 7), favoring the use of ESWT combined with CDT rather than CDT on its own.

The pathophysiology of lymphedema has become a vital issue in the medical community. The accumulation of protein-rich interstitial fluid hinders the lymphatic function, leading to heaviness, tightness, and chronic inflammation. These pathological changes promote tissue fibrosis and decrease lymphangiogenesis. The increase in cytokines also inhibits Lymphatic Endothelial Cell (LEC) proliferation and tubule formation, migration, and function [45]. Considering the mechanism resulting in the benefits of ESWT on BCRL, we have concluded several possible theories as follows. First, stretching the skin gives tension to the anchoring filaments, which pulls the LEC and opens the junctions between LEC. As the junctions open, the accumulated fluid can enter the lymphatic lumen and be collected [46]. We supposed that ESWT might have a similar stretching effect and thus improve the lymphatic drainage. Besides, several studies have discovered that ESWT reduces skin fibrosis [47]. These findings may also affect the fibrosis caused by the interstitial fluid accumulation in BCRL patients. Finally, ESWT affects the molecular aspects of lymphangiogenesis. Serizawa et al. created a rat model of tail lymphedema, showing up-regulation of VEFG-C in groups treated with ESWT [48]. In another study by Kubo et al., the author demonstrated an elevation of VEGF receptor 3 expression in rabbit ear treated with ESWT [23]. These effects may contribute to increased lymphangiogenesis, which improves the symptoms of BCRL.

Limited ROM was frequently reported due to BCRL, yet other factors such as surgical procedure and radiotherapy could also lead to a stiffened shoulder [49,50]. This occurrence could possibly be explained by subcutaneous scarring or connective tissue formation. For example, axillary web syndrome (AWS) is another common complication following breast cancer surgery. The cord-like connective tissue affected more than half of the patients receiving axillary lymph node dissection, causing pain, limited function, and impaired shoulder ROM [51,52]. Since we had identified that ESWT combined with CDT improved shoulder joint ROM in patients with BCRL, we assumed that ESWT could also be applied to patients with AWS. However, further studies are still required to confirm this hypothesis.

Based upon the latest guidelines regarding rehabilitation intervention for BCRL, CDT is the standard treatment for stage two and three (moderate-to-severe) patients [53]. Likewise, while most of our assessed studies explored the effect of ESWT in addition to CDT, only one (Cebicci et al., 2021 [43]) compared the effectiveness of ESWT with CDT. Being a relatively novel treatment, strong evidence for using ESWT rather than CDT is still lacking. As a result, we suggest seeing ESWT as a form of supplementary intervention to CDT before more research emerges.

All the assessed studies reported the immediate or short-term effect of ESWT, while long-term follow-up was lacking. Since lymphedema is considered a lifelong problem, it is important to follow up and compare the long-term effect of ESWT in patients with BCRL. Another noteworthy issue is the impact of ESWT on malignant cell regeneration. Malignant tumors and metastasis in the treatment area are considered contraindications for ESWT [54]. Although no adverse effects had been reported in those reviewed studies, concerning ESWT’s positive effect on endothelial neoangiogenesis and tissue growth, evaluation of both its safety and influence on breast cancer survivors is crucial.

### Limitations

First, there were only a few available studies regarding the use of ESWT in patients with BCRL. The sample size was small, and the overall quality of evidence was quite low. Although meta-analysis could increase confidence and the level of evidence, results are inevitably influenced by the biases in the assessed trials. Nevertheless, we summarized the BCRL outcome parameters and ESWT treatment protocols used in the previous studies, which may help in designing further clinical trial of related topics. With more high-quality trials being performed, we may draw a more solid conclusion regarding the effects of ESWT on BCRL.

Second, variations in ESWT parameters among the studies were present, including the ESWT mode (focused or radial), treatment area, treatment frequency, and dosage. The heterogeneity restricted our attempt to determine an optimal therapeutic protocol. Additionally, the timing of intervention is also an import issue for patients and clinicians. None of the selected studies explored the relation between the duration of BCRL and the effectiveness of ESWT. Considering the expanding demand for more safe and efficient interventions for BCRL, the establishment of a standard ESWT protocol is thus imperative.

## 5. Conclusions

In summary, ESWT appears to have positive effects on certain BCRL symptoms. The combined use of ESWT and CDT could significantly improve the volume of lymphedema, skin thickness, and shoulder ROM as compared to CDT on its own. However, current evidence for these benefits is still of low methodological quality. In addition, there is still not enough evidence to support the use of ESWT as a replacement for CDT either.

## Figures and Tables

**Table 1:** Inclusion and exclusion criteria.

	Inclusion	Exclusion
Design	Randomized controlled trials Cohort studies Observational studies	Preclinical study
Participants	People with Breast-Cancer-Related Lymphedema	Lymphedema not related to breast cancer surgery
Intervention	Extracorporeal shockwave therapy with or without other management	
Outcomes measures	Primary: volume or arm circumference Secondary: shoulder joint ROM, other quantification of symptoms, impact on QOL	
Language	English or Chinese with an abstract in English	Other languages

Abbreviations: ROM, range of motion; QOL, quality of life.

**Table 2:** Patients’ characteristics of included studies.

Study	Design	Participants (Stage of LE)	No. of Patient (ESWT/Control Group)	Age (Mean ± SD, Year)	LE Duration (Mean ± SD, Month)
Mahran et al., 2015 (Egypt) [38]	RCT	BCRL (N/A)	ESWT + CDT: 20 CDT: 20	52.13 ± 4.0 53.80 ± 3.4	15.60 ± 2.82 14.73 ± 2.86
El-Shazly et al., 2016 (Egypt) [39]	RCT	BCRL (Stage 2, 3)	ESWT + CDT: 30 CDT: 30	30–50 (Only range)	N/A
El-Shazly et al., 2016 (Egypt) [40]	RCT	BCRL (Stage 2, 3)	ESWT + CDT: 30 CDT: 30	30–50 (Only range)	N/A
Abdelhalim et al., 2018 (Egypt) [41]	RCT	BCRL (N/A)	ESWT + CDT: 21 CDT: 22	48.71 ± 3.07 49.55 ± 2.77	10.95 ± 1.59 11.17 ± 1.61
Lee et al., 2020 (South Korea) [42]	RCT	BCRL (Stage 2)	ESWT + CDT: 15 CDT: 15	53.13 ± 10.85 52.24 ± 8.60	12.83 ± 8.21 14.40 ± 10.63
Cebicci et al., 2021 (Turkey) [43]	RCT	BCRL (N/A)	ESWT: 10 CDT: 10	51.61 ± 6.6 57.90 ± 6.9	32.7 ± 31.1 31.6 ± 30.0
Cebicci et al., 2016 (Turkey) [27]	Prospective pilot study	BCRL (N/A)	ESWT: 11	50.63 ± 7.03	12 (Range: 6–84)
Joos et al., 2020 (Belgium) [44]	Prospective pilot study	BCRL (Stage 3)	ESWT + CDT: 10	62.1 ± 8.21	61.9 ± 17.55

Abbreviations: LE: Lymphedema; No.: Number; SD: Standard deviation; RCT: Randomized controlled trial; BCRL: Breast cancer-related lymphedema; N/A: not available; ESWT: Extracorporeal shock wave therapy; CDT: Complex decongestive therapy.

**Table 3:** Therapeutic protocols of included studies.

Study	Type of ESWT	ED (mJ/mm^2^)	Impulse Frequency	Dosage and Location		Interval and Sessions
Mahran et al., 2015 (Egypt) [38]	rESWT	2 bar	4 Hz	750 250 1500	Axillary LN Cubital LN Arm, Forearm, Hand	2/wk for 16 sessions
El-Shazly et al., 2016 (Egypt) [39]	fEWST	0.040–0.069	5 Hz	1000 1000	Most fibrotic tissue Lesser fibrotic tissue	2/wk for 12 sessions
El-Shazly et al., 2016 (Egypt) [40]	fEWST	0.040–0.069	4 Hz	1000 1000	Most fibrotic tissue Lesser fibrotic tissue	2/wk for 12 sessions
Abdelhalim et al., 2018 (Egypt) [41]	rESWT	2 bar	4 Hz	750 250 1500	Axillary LN Cubital LN Arm, Forearm, Hand	3/wk for 12 sessions
Lee et al., 2020 (South Korea) [42]	fEWST	0.056–0.068	NA	1000 1500	Most fibrotic areaCubital LN, forearm	2/wk for 6 sessions
Cebicci et al., 2021 (Turkey) [43]	rESWT	2 bar	4 Hz	750 250 1500	Axillary LN Cubital LN Arm, Forearm, Hand	3/wk for 12 sessions
Cebicci et al., 2016 (Turkey) [27]	rESWT	2 bar	4 Hz	750 250 1500	Axillary LN Cubital LN Arm, Forearm, Hand	3/wk for 12 sessions
Joos et al., 2020 (Belgium) [44]	fEWST	0.1	4 Hz	1800 800	Most fibrotic area Grid pattern around this area	2/wk for 8 sessions

Abbreviations: ESWT: Extracorporeal shock wave therapy; ED: Energy flux density; rESWT: Radial extracorporeal shock wave therapy; LN: Lymph node;/wk: per week; fESWT: Focused extracorporeal shock wave therapy; N/A: not available.

**Table 4:** Different methods were used to quantify symptoms of Breast cancer-related lymphedema in included trials.

Study	Study Type	Volume	Arm Circumference	Skin Thickness	qDASH	ROM
Mahran et al., 2015 (Egypt) [38]	RCT	Yes	Yes	No	No	Yes
El-Shazly et al., 2016 (Egypt) [39]	RCT	Yes	No	No	No	Yes
El-Shazly et al., 2016 (Egypt) [40]	RCT	No	No	Yes	No	No
Abdelhalim et al., 2018 (Egypt) [41]	RCT	No	Yes	Yes	No	No
Lee et al., 2020 (South Korea) [42]	RCT	Yes	Yes	Yes	Yes	No
Cebicci et al., 2021 (Turkey) [43]	RCT	Yes	Yes	No	Yes	No
Cebicci et al., 2016 (Turkey) [27]	Prospective pilot study	Yes	No	No	Yes	No
Joos et al., 2020 (Belgium) [44]	Prospective pilot study	Yes	Yes	No	No	No

Abbreviations: RCT: Randomized controlled trial; qDASH: quick Disabilities of the Arm, Shoulder, and Hand; ROM: Range of motion.

**Table 5:** Outcome measurements of the volume of included studies for ESWT in BCRL.

Study	Design	No. of Patients (ESWT/Control Group)	Measurement	Volume (Mean ± SD, mL)		Intragroup Difference	Intergroup Difference
				Baseline	Post-Intervention		
Mahran et al., 2015 (Egypt) [38]	RCT	ESWT + CDT: 20	Difference of healthy and affected arm (15 cm above olecranon)	811.9 ± 68.18	285.6 ± 30.06	Yes	Yes
		CDT: 20		797.7 ± 80.33	363.7 ± 24.40	Yes	
El-Shazly et al., 2016 (Egypt) [39]	RCT	ESWT + CDT: 30	Affected arm (N/A)	1219.33 ± 83.42	924.04 ± 94.71	Yes	Yes
		CDT: 30		1235.40 ± 84.12	1043.85 ± 90.32	Yes	
Lee et al., 2020 (South Korea) [42]	RCT	ESWT + CDT: 15	Difference of healthy and affected arm (axillary level)	840.42 ± 181.33	802.80 ± 149.70	Yes	Yes
		CDT: 15		822.00 ± 144.68	810.00 ± 156.90	No	
Cebicci et al., 2021 (Turkey) [43]	RCT	ESWT: 10	Difference of healthy and affected arm (axillary level)	932.0 ± 341.9	795.0 ± 360.9	Yes	No
		CDT: 10		800.0 ± 402.7	675.0 ± 345.8	Yes	
Cebicci et al., 2016 (Turkey) [27]	Prospective pilot study	ESWT: 11	Difference of healthy and affected arm (axillary level)	870.4 ± 115.1	736.36 ± N/A	Yes	N/A
Joos et al., 2020 (Belgium) [44]	Prospective pilot study	ESWT + CDT: 10	Affected arm (N/A)	3086.4 ± 539.47	2909.1 ± 471.60	No	N/A

Abbreviations: ESWT: Extracorporeal shock wave therapy; BCRL: Breast cancer-related lymphedema; No.: Number; SD: Standard deviation; mL: milliliter; RCT: Randomized controlled trial; CDT: Complex decongestive therapy; N/A: Not available.

**Table 6:** Outcome measurements of the arm circumference in included studies for ESWT in BCRL.

Study	Design	No. of Patients (ESWT/Control Group)	Measurement		Intragroup Difference	Intergroup Difference
Mahran et al., 2015 (Egypt) [38]	RCT	ESWT + CDT: 20	Total circumferential differences	20 cm AE 15 cm AE 10 cm AE 10 cm BE 15 cm BE 20 cm BE	Yes	Yes
		CDT: 20			Yes	
Abdelhalim et al., 2018 (Egypt) [41]	RCT	ESWT + CDT: 30	Difference of healthy and affected arm	10 cm below axilla 10 cm AE 7 cm BE 7 cm above wrist	Yes	Yes
		CDT: 30			Yes	
Lee et al., 2020 (South Korea) [42]	RCT	ESWT + CDT: 15	Affected arm	10 cm AE Elbow 10 cm BE Wrist MCP	Yes (only 10 cm BE)	No
		CDT: 15			No	
Cebicci et al., 2021 (Turkey) [43]	RCT	ESWT: 10	Difference of healthy and affected arm	15 cm AE 10 cm BE Wrist MCP	Yes	No
		CDT: 10			Yes (only 10 cm BE and wrist)	
Joos et al., 2020 (Belgium) [44]	Prospectivepilot study	ESWT + CDT: 10	Affected arm	10 cm AE Elbow 10 cm BE	No	N/A

Abbreviations: ESWT: Extracorporeal shock wave therapy; BCRL: Breast cancer-related lymphedema; No.: Number; RCT: Randomized controlled trial; CDT: Complex decongestive therapy; cm: centimeter; AE: Above elbow; BE: Below elbow; MCP: Netacarpophalangeal joint; N/A: Not available.

## Data Availability

The data presented in the study were obtained from the included trials and are openly available.

## Supplementary Materials

The followings are available online at https://www.mdpi.com/article/10.3390/jcm10245970/s1, Table S1: Search strategy; Table S2: Reasons for exclusion and reference of excluded studies; Table S3: Studies and methodological quality description according to the PEDro Scale Score; Table S4: Studies and methodological quality description according to the Modified Jadad Scale with eight items; Table S5: Summary of quality assessment (using STROBE assessment tools) of the included articles; Table S6: Appraisal of included studies, using the GRADE tool; Table S7: Skin thickness difference measurement of included studies; Table S8: ROM measurement of included studies; Table S9: Arm circumference measurement of included studies; Table S10: qDASH measurement of included studies.
