# Supplementary material for: Extracorporeal Shock Wave Therapy Combined with Complex Decongestive Therapy in Patients with Breast Cancer-Related Lymphedema: A Systemic Review and Meta-Analysis
Source: J Clin Med. 2021 Dec 19;10(24):5970. doi: 10.3390/jcm10245970 (PMC8705697; doi:10.3390/jcm10245970)
Supplement: Supplementary file 1 [file jcm-10-05970-s001.zip › jcm-1518350-supplementary.pdf]

Table S1. Search strategy.

**Appendix 1a. PubMed**<br>((((("Extracorporeal Shockwave Therapy"[Mesh]) NOT "Lithotripsy"[Mesh]) AND "Lymphedema"[Mesh]) OR "Non-Filarial Lymphedema"[Mesh]) AND "Breast Neoplasms"[Mesh]) OR "Breast Cancer Lymphedema"[Mesh]
**Appendix 1b. PEDro**<br>Abstract and Title: shockwave<br>AND<br>Problem: lymphedema<br>AND<br>Body Part: upper arm, shoulder, or shoulder girdle<br>AND<br>Subdiscipline: oncology<br>AND<br>Method: clinical trial
**Appendix 1c. Embase**<br>#1 'breast tumor' AND 'lymphedema therapy': ab,ti AND 'shock wave therapy': ab,ti<br>#2 'breast tumor' AND 'lymphedema': ab,ti AND 'shock wave therapy': ab,ti<br>#3 'breast tumor' AND 'lymphedema' AND 'shock wave therapy'<br>#4 breast AND cancer AND lymphedema AND shockwave<br>#5 #1 AND #2 AND #3 AND #4<br>#6 'breast cancer' AND lymphedema AND shock AND wave
**Appendix 1d. Cochrane Library Databases**<br>Title Abstract Keywords:<br>#1 shockwave AND lymphedema AND breast cancer<br>#2 ESWT AND lymphedema AND breast cancer<br>#3 shockwave AND lymphedema<br>#4 ESWT AND lymphedema
**Appendix 1e. Google Scholar**<br>'ESWT' AND 'lymphedema'Table S1; AND 'breast cancer'

Table S2a. Reasons for exclusion.(n = 13)

Study	Decision in manuscript	Additional notes
Kim et al. 2015	Case report/serials	Computed tomography as an objective measurement tool for secondary lymphedema in two patients
Ha et al. 2021	Case report/serials	Case report in Korea
Michelini et al. 2007	Lymphedema not related to breast cancer	ESWT on secondary lymphedema
Michelini et al. 2010	Lymphedema not related to breast cancer	ESWT on geriatrics lymphedema
Kim et al. 2013	Pre-clinical study	Animal study (mouse)
Rohringer et al. 2014	Pre-clinical study	Molecular and cellular effects of shockwave treatment on in vitro lymphatic endothelial cells
Schaupper et al. 2016	Pre-clinical study	Preclinical review of lymphatic vessels in regenerative medicine and tissue engineering
Alderfer et al. 2018	Pre-clinical study	Preclinical review of lymphatic tissue engineering and regeneration
Asaad et al. 2021	Pre-clinical study	Preclinical review of tissue engineering strategies for Cancer-Related Lymphedema
Cho et al. 2021	Pre-clinical study	Animal study (rat)
Miccinilli et al. 2020	Literature review	A literature review for ESWT on BCRL
Konrad et al. 2021	Literature review	Narrative review of in vitro, animal, and clinical studies
Bae et al. 2013	Inappropriate study design	Outcomes of ESWT group and ESWT+CDT group were calculated together; outcome measurement like this was not appropriate

Abbreviations: ESWT: Extracorporeal Shock Wave Therapy; BCRL: Breast-Cancer-Related Lymphedema; CDT: Complex Decongestive Therapy.

Table S2b. Reference of excluded studies.(n = 13)

1. Kim, S.Y.; Bae, H.; Ji, H.M. Computed Tomography as an Objective Measurement Tool for Secondary Lymphedema Treated With Extracorporeal Shock Wave Therapy. *Ann. Rehabil. Med*. **2015**, *39*, 488-493.
2. Ha, M.C.; Shin, J.C.; Jung, Y.S.; Im, S.H. Feasibility of Extracorporeal Shock Wave Therapy for Complex Upper Limb Morbidity in Breast Cancer Patient. *Clin. Pain*. **2021**, *20*, 25-29.
3. Michelini, S.; Failla, A.; Moneta, G.; Zinicola, V.; Macaluso, B.; Cardone, M.; Antonucci, D. Treatment of lymphedema with shockwave therapy: preliminary study. *Eur. J. Lymphology Relat. Probl.* **2007**, *17*, 29.
4. Michelini, S.; Cardone, M.; Failla, A.; Moneta, G.; Fiorentino, A.; Cappellino, F. Treatment of geriatrics lymphedema with shockwave therapy. *BMC Geriatrics*. **2010**;*10(Suppl 1)*:A105.
5. Kim, I.G.; Lee, J.Y.; Lee, D.S.; Kwon, J.Y.; Hwang, J.H. Extracorporeal shock wave therapy combined with vascular endothelial growth factor-C hydrogel for lymphangiogenesis. *J. Vasc. Res*. **2013**, *50*, 124-133.
6. Rohringer, S.; Holnthoner, W.; Hackl, M.; Weihs, A.M.; Rünzler, D.; Skalicky, S.; Karbiener, M.; Scheideler, M.; Pröll, J.; Gabriel, C.; Schweighofer, B.; Gröger, M.; Spittler, A.; Grillari, J.; Redl, H. Molecular and cellular effects of in vitro shockwave treatment on lymphatic endothelial cells. *PLoS One*. **2014**, *9*, e114806.
7. Schaupper, M.; Jeltsch, M.; Rohringer, S.; Redl, H.; Holnthoner, W. Lymphatic Vessels in Regenerative Medicine and Tissue Engineering. *Tissue Eng. Part B Rev.* **2016**, *22*, 395-407.
8. Alderfer, L.; Wei, A.; Hanjaya-Putra, D. Lymphatic Tissue Engineering and Regeneration. *J. Biol. Eng.* **2018**, *12*, 32.
9. Asaad, M.; Hanson, S.E. Tissue Engineering Strategies for Cancer-Related Lymphedema. *Tissue Eng. Part A*. **2021**, *27*, 489-499.
10. Cho, H.K.; Sung, W.J.; Lee, Y.J.; Kwak, S.G.; Kim, K.L. Two methods of extracorporeal shock-wave therapy in a rat model of secondary lymphedema: A pilot study. *J. Int. Med. Res.* **2021**, *49*, 3000605211024473.
11. Miccinilli, S.; Bravi, M.; Maselli, M.; Santacaterina, F.; Morrone, M.; Manco, D.; Toglia, R.; Sterzi, S.; Bressi, F. The effectiveness of extracorporeal shock wave therapy on breast cancer-related lymphedema: A literature review. *Lymphology*. **2020**, *53*, 118-135.
12. Konrad, J.; Grygiel, M. Extracorporeal shock wave therapy as a potential therapeutic tool for breast cancer related lymphedema: A narrative review of intro, animal and clinical studies. *Med. Sci. Pulse.* **2021**, *15*, 66–73.

13. Bae, H.; Kim, H.J. Clinical outcomes of extracorporeal shock wave therapy in patients with secondary lymphedema: A pilot study. *Ann. Rehabil. Med*. **2013**, *37*, 229-234.

Table S3. Studies and methodological quality description according to the PEDro Scale Score.

Study	Random allocation	Concealed allocation	Groups similar at baseline	Participant blinding	Therapist blinding	Assessor blinding	< 15% dropouts	Intention -to-treat analysis	Between-group difference reported	Point estimate and variability reported	Total (0 to 10)
Mahran et al. 2015 [38]	Y	N	Y	N	N	N	N	N	Y	Y	4
El-Shazly et al. 2016 [39]	Y	N	Y	N	N	N	N	N	Y	Y	4
El-Shazly et al. 2016 [40]	Y	N	Y	N	N	N	N	N	Y	Y	4
Abdelhalim et al. 2018 [41]	Y	Y	Y	N	N	Y	Y	N	Y	Y	7
Lee et al. 2020 [42]	Y	N	Y	N	N	N	Y	Y	Y	Y	6
Cebicci et al. 2021 [43]	Y	Y	Y	N	N	N	N	N	Y	Y	5

Table S4. Studies and methodological quality description according to the Modified Jadad Scale with eight items.

**Study**	Mahran et al. 2015 [38]	El-Shazly et al. 2016 [39]	El-Shazly et al. 2016 [40]	Abdelhalim et al. 2018 [41]	Lee et al. 2020 [42]	Cebicci et al. 2021 [43]
Was the study described as randomized?	Yes	Yes	Yes	Yes	Yes	Yes
Was the method of randomization appropriate?	Not described	Not described	Not described	Yes	Not described	Yes
Was the study described as blinded?	No	No	No	Yes[a]	No	No
Was the method of blinding appropriate?	No	No	No	Yes	No	No
Was there a description of withdrawals and dropouts?	No	No	No	Yes	Yes	Yes
Was there a clear description of the inclusion/exclusion criteria?	Yes	Yes	Yes	Yes	Yes	Yes
Was the method used to assess adverse effects described?	No	No	No	No	Yes	No
Was the method of statistical analysis described?	Yes	Yes	Yes	Yes	Yes	Yes
Total	3	3	3	6.5	5	5

[a] Single-blind.

Table S5. Summary of quality assessment (using STROBE assessment tools) of the included articles.

Report section	Item		Cebicci et al. 2016 [27]	Joos et al. 2020 [44]
**Title and abstract**	Title	1a	Y	Y
	Abstract	1b	Y	Y
**Introduction**	Background/rationale	2	Y	Y
	Objective	3	N	Y
**Methods**	Study design	4	Y	Y
	Setting(location + dates)	5	Y	N
	Participants	6a	Y	Y
		6b	N/A[a]	N/A[a]
	Variables	7	Y	Y
	Data (sources/measurement)	8	Y	Y
	Bias	9	N	N
	Study size	10	N	N
	Quantitative variables	11	Y	Y
	Statistical methods	12a	Y	Y
		12b	N	N
		12c	N	N
		12d	N	N
		12e	N	N
**Results**	Participants	13a	Y	Y
		13b	Y[b]	Y
		13c	Y	N
	Descriptive data	14a	Y	Y
		14b	Y[b]	N
		14c	Y	Y
	Outcome data	15	Y	Y
	Main results	16a	N	Y
		16b	N/A[a]	N/A[a]
		16c	N	N
	Other analyses	17	N	N
**Discussion**	Key results	18	Y	Y
	Limitations	19	Y	Y
	Interpretation	20	Y	Y
	Generalisability	21	Y	Y
**Other**	Funding	22	N	Y
**Total percentage for all sub-items**			65.6%(21/32)	65.6%(21/32)

Abbreviations: STROBE: Strengthening the Reporting of Observational studies in Epidemiology; N/A: Not Assessed; %: Percentage.

[a] N/A was not calculated for percentage.

[b] No patients withdrew from this study.

Table S6. Appraisal of included studies, using the GRADE tool.

Quality assessment							Quality of evidence
No. of studies	Design	Risk of bias	Inconsistency	Indirectness	Imprecision	Other considerations	
Volume of lymphedema[a](ESWT combined with CDT versus CDT)							
2 (70 patients)	RCT	Serious[b]	No serious inconsistency	No serious indirectness	Serious[c]	Publication bias[d] Intervention variaton[e]	♁○○○ Very low
Skin thickness difference(ESWT combined with CDT versus CDT)							
2 (73 patients)	RCT	Serious[b]	No serious inconsistency	No serious indirectness	Serious[c]	Publication bias[d] Intervention variaton[e]	♁○○○ Very low
Range of motion – shoulder (ESWT combined with CDT versus CDT)							
2 (100 patients)	RCT	Serious[b]	No serious inconsistency	No serious indirectness	Serious[c]	Publication bias[d] Intervention variaton[e]	♁○○○ Very low

[a] Volume of the lymphedema was measured by the water-displacement method; the volume difference between the affected limb and healthy limb was calculated.

[b] Some studies had a high risk of bias due to their methodology.

[c] Studies with a small sample size.

[d] Only two studies meet the criteria for analysis.

[e] Type of Extracorporeal Shock Wave Therapy and therapeutic sessions varied across studies.

[f] Difference of the skin thickness was measured at 7cm below the elbow in the study of Abdelhalim et al. [41] and at 10cm below the elbow in the study ofLee et al. [42].

[g] $I^2 = 86\%$, considerable heterogeneity.

[h] $I^2 = 84\%$, considerable heterogeneity.

Abbreviations: GRADE: The Grading of Recommendations Assessment, Development and Evaluation; No.: number; ESWT: Extracorporeal Shock Wave Therapy; CDT: Complex Decongestive Therapy; RCT: Randomized Control Trial.

Table S7. Skin thickness difference measurement of included studies.

Study	No. of patients	Measurement	Difference of the skin thickness (mean±SD, mm)		Intragroup difference	Intergroup difference
			Baseline	Post-intervention		
El-Shazly et al. 2016 (Egypt) [40]	ESWT+CDT: 30<br>CDT: 30	Sonography (most fibrotic point)	2.31±0.55	1.79±0.46	Yes	Yes
			2.33±0.57	2.3±0.59	No	
Abdelhalim et al. 2018 (Egypt) [41]	ESWT+CDT: 21<br>CDT: 22	Skinfold caliper[a]	22.81±1.12	16.43±3.11	Yes	Yes
			23.05±1.00	18.50±2.87	Yes	
Lee et al. 2020 (South Korea) [42]	ESWT+CDT: 15<br>CDT: 15	Skinfold caliper[a]	31.14±2.91	29.85±3.09	Yes	Yes
			30.15±7.40	29.54±6.98	No	

[a] Difference of the skin thickness was measured at 7cm below the elbow in studies of Abdelhalim et al. [41] and Bae et al. [26], and at 10cm below the elbow in study of Lee et al. [42].

Abbreviations: No.: number; SD: Standard Deviation; mm: millimeter; ESWT: Extracorporeal Shock Wave Therapy; CDT: Complex Decongestive Therapy.

Table S8. ROM measurement of included studies.

Study	No. of patients	Flexion (mean±SD, degree)		Abd (mean±SD, degree)		ER (mean±SD, degree)	
		Baseline	Post-intervention	Baseline	Post-intervention	Baseline	Post-intervention
Mahran et al. 2015 (Egypt) [38]	ESWT+CDT: 20<br>CDT: 20	154.7±9.87<br>156.9±6.70	174.44±6.70<br>166.80±6.66	160.2±7.37<br>162.2±6.00	178.0±5.88<br>172.9±4.65	72.1±3.68<br>71.9±4.86	85.9±2.23<br>82.2±3.85
El-Shazly et al. 2016 (Egypt) [39]	ESWT+CDT: 30<br>CDT: 30	95.12±7.81<br>97.30±11.60	120.44±13.07<br>111.83±13.91	82.13±11.20<br>80.80±12.38	114.10±14.62<br>97.66±15.70	31.51±3.68<br>30.30±5.12	42.93±5.04<br>35.20±4.62

Abbreviations: ROM: Range Of Motion; No.: number; SD: Standard Deviation; Abd: Abduction; ER: External Rotation; ESWT: Extracorporeal Shock Wave Therapy; CDT: Complex Decongestive Therapy.

Table S9. Arm circumference measurement of included studies.

Study	No. of patients	Upper arm (cm)		Elbow (cm)		Forearm (cm)		Wrist (cm)	
		Baseline	Post Tx	Baseline	Post Tx	Baseline	Post Tx	Baseline	Post Tx
Abdelhalim et al. 2018 (Egypt) [41]	ESWT+CDT: 30	6.405	4.2	N/A	N/A	6.165	2.3445	N/A	N/A
	CDT: 30	6.76	6.725	N/A	N/A	5.79	4.885	N/A	N/A
Lee et al. 2020 (South Korea) [42]	ESWT+CDT: 15	28.17	27.14	25.94	25.63	26.28	25.50	16.21	16.00
	CDT: 15	26.81	25.55	24.44	24.04	25.80	25.40	16.70	16.55
Cebicci et al. 2021 (Turkey) [43]	ESWT: 10	3.4	2.7	N/A	N/A	3.7	2.7	1.7	1.3
	CDT: 10	2.6	1.9	N/A	N/A	3.9	2.9	1.8	1.6
Joos et al. 2020 (Belgium) [44]	ESWT+CDT: 10	32.3	31.4	29.1	28.1	27.50	26.80	N/A	N/A

Abdelhalim et al. [41] and Cebicci et al. [43] was calculated the difference of the healthy and affected arm, while Lee et al. [42] and Joos et al. [44] measured the arm circumference of affected side only.

Abbreviations: No.: number; cm: centimeter; Tx: Treatment; ESWT: Extracorporeal Shock Wave Therapy; CDT: Complex Decongestive Therapy; N/A: not available..

Table S10. qDASH measurement of included studies.

Study	No. of patients	qDASH score (±SD)		Intragroup difference	Intergroup difference
		Baseline	Post-intervention		
Lee et al. 2020 (South Korea) [42]	ESWT+CDT: 15	4.25±5.72	3.89±4.41	No	No
	CDT: 15	3.15±4.28	3.11±3.98	No	
Cebicci et al. 2021 (Turkey) [43]	ESWT: 10	72.4±16.6	63.6±14	Yes	Yes
	CDT: 10	74.9±10.1	70.7±9.6	Yes	
Cebicci et al. 2016 (Turkey) [27]	ESWT: 11	70.65±N/A	62.18±N/A	Yes	N/A

Abbreviations: No.: number; qDASH: quick Disabilities of the Arm, Shoulder, and Hand; SD: Standard Deviation; ESWT: Extracorporeal Shock Wave Therapy; CDT: Complex Decongestive Therapy; N/A: not available.
